# Maturation of human cardiomyocytes derived from induced pluripotent stem cells (iPSC-CMs) on polycaprolactone and polyurethane nanofibrous mats

**DOI:** 10.1038/s41598-024-63905-z

**Published:** 2024-06-05

**Authors:** Zuzanna Iwoń, Ewelina Krogulec, Inez Tarnowska, Iwona Łopianiak, Michał Wojasiński, Agnieszka Dobrzyń, Elżbieta Jastrzębska

**Affiliations:** 1 Chair of Medical Biotechnology, Faculty of Chemistry, Warsaw University of Technology, Noakowskiego 3, 00-664 Warsaw, Poland; 2 Laboratory of Cell Signaling and Metabolic Disorders, Nencki Institute of Experimental Biology PAS, Warsaw, Poland; 3 Department of Biotechnology and Bioprocess Engineering, Faculty of Chemical and Process Engineering, Warsaw University of Technology, Warsaw, Poland; 4 Centre for Advanced Materials and Technologies, CEZAMAT Warsaw University of Technology, Warsaw, Poland

**Keywords:** Cell biology, Cardiology, Biomedical engineering

## Abstract

Investigating the potential of human cardiomyocytes derived from induced pluripotent stem cells (iPSC-CMs) in in vitro heart models is essential to develop cardiac regenerative medicine. iPSC-CMs are immature with a fetal-like phenotype relative to cardiomyocytes in vivo. Literature indicates methods for enhancing the structural maturity of iPSC-CMs. Among these strategies, nanofibrous scaffolds offer more accurate mimicry of the functioning of cardiac tissue structures in the human body. However, further research is needed on the use of nanofibrous mats to understand their effects on iPSC-CMs. Our research aimed to evaluate the suitability of poly(ε-caprolactone) (PCL) and polyurethane (PU) nanofibrous mats with different elasticities as materials for the maturation of iPSC-CMs. Analysis of cell morphology and orientation and the expression levels of selected genes and proteins were performed to determine the effect of the type of nanofibrous mats on the maturation of iPSC-CMs after long-term (10-day) culture. Understanding the impact of 3D structural properties in in vitro cardiac models on induced pluripotent stem cell-derived cardiomyocyte maturation is crucial for advancing cardiac tissue engineering and regenerative medicine because it can help optimize conditions for obtaining more mature and functional human cardiomyocytes.

## Introduction

Cardiovascular diseases (CVDs) cause millions of deaths worldwide every year^1^. Such a high mortality rate is associated with the poor regenerative capacity of cardiomyocytes (CMs) belonging to the cells of the myocardial cells^2^. Current treatments to support the work of the heart are based on medications and surgery. However, they do not lead to the complete regeneration of the damaged tissue^3^. The study of various methods of regenerating the cardiac muscle initially requires obtaining cellular models composed of mature human heart cells, which are very difficult to obtain. Using in vitro models with mature heart cells will make it possible to understand and explain the processes occurring in the cardiac tissue. The cellular models used so far use immature heart cells with a different morphology and physiology than mature heart cells and, therefore, do not adequately mimic the in vivo conditions of adult cardiac tissue^4^. Currently, scientists are interested in the use of human cardiomyocytes differentiated from induced pluripotent stem cells (iPSC-CMs) for in vitro models^4–6^.

Nowadays, procedures are known to obtain cardiomyocytes from iPSCs after differentiation with high efficiency (80–98% of the population undergoes differentiation)^7,8^. However, hiPSC-CMs are cardiac cells whose physiology, morphology, and functionality are more like fetal human cardiomyocytes than mature cells^9–11^. For example, adult cardiomyocytes can have two or more cell nuclei, an anisotropic, rod-shaped shape, about 150 μm^2^ in area. In contrast, fetal CMs and iPSC-CMs have mostly one cell nucleus, do not show an anisotropic, elongated shape, and are smaller than adult human CMs (iPSC-CMs have about 30 μm^2^, and the area of fetal CMs is smaller than adult cardiomyocytes)^10^. Also, the structure of sarcomeres, electrophysiology, and metabolism of iPSC-CMs are similar to fetal cardiac cells than adult CMs^11,12^. Therefore, it is necessary to use, for example, physical factors such as nanofibers^13,14^ or biochemical factors such as growth factors^15^ to carry out the maturation process. Using nanofibers in vitro cellular models of the heart makes it possible to obtain scaffolds that mimic the mechanical and structural properties of the cardiac extracellular matrix in vivo. The extracellular matrix (ECM) in cardiac tissue supports myocardial contraction (sarcomeres and myofibrils), helps in cell-to-cell communication, and affects the morphology and physiology of cardiac tissue^16^. The literature shows that PCL and PU nanofibrous mats are commonly utilized in 3D cardiac cultures^17–21^. However, there is a lack of comparison on whether nanofibers' physicochemical properties (polymer type, elasticity) affect the functioning of cardiac cells. PCL and PU nanofibrous mats are biocompatible, biodegradable, and have accurate mechanical strength and elasticity to culture cardiac cells^22,23^. However, both polymers are hydrophobic; therefore, modifying the surface with oxygen plasma and protein solution such as gelatin or collagen is necessary. For example, Safavian et al. fabricated PCL nanofibers, and the surface of the nanofibrous mats was modified with oxygen plasma to increase their hydrophilicity. Then, adipose-derived stem cells (ASCs) were seeded on the randomly and parallel-oriented nanofibers. The cells were differentiated into cardiomyocytes for 7, 14, and 21 days of culture. Cell viability analysis was performed using the MTT assay, and it was determined that cells cultured on arrangement nanofibrous mats had higher viability than cells cultured on random nanofibers. It was found that ACS cells cultured on nanofibers had an elongated, rod-like shape which is characteristic of cardiomyocytes. Increased expression of cardiac tissue marker genes was also observed, including troponin T, α-MHC, and GATA-4. The expression of these genes was higher in cells cultured on parallel arrangement nanofibers^24^. Additionally, recent literature sources indicate that various nanofibers affect the arrangement, electrophysiology, and structural maturation of iPSC-CMs^13,14,25^. For example, Ding et al*.* compared differentiation (iPSC)-derived cardiac progenitor cells (CPCs) into cardiomyocytes on 2D (culture in polystyrene plate) and 3D models (grown on PCL nanofibers). They noticed a significant increase in cardiac-specific genes such as *MYH7* and *ACTA2* and synchronized intracellular Ca^2+^ oscillation similar to adult cardiomyocytes^14^. In another work, nanofibrous mats made of polylactide-glycolic acid (PLGA) with parallel orientation were used to study iPSCs-CM maturation. It was confirmed that cultures grown on nanofibrous mats have a more elongated, anisotropic morphology. In addition, there was an increase in the expression of proteins that are markers of cardiomyocyte maturity (troponin T, α-actinin) and proteins that play a major role in myocardial contraction (β-MHC), after 14 days of culture, compared to the control culture on a polystyrene plate (PS). In addition, an increase in the expression of structural genes (*ACTN2* and *TNNI3*), cardiac maturation gene (*MYH7*), and genes related to the regulation of Ca^2+^ ion release (*PLN* and *RYR2*) were observed^25^.

Research into the potential use of iPSC-CMs in cardiac cell in vitro models is an important element for the development of regenerative medicine. Even though cardiomyocytes differentiated from iPSCs have features that distinguish them from native cells from adult cardiac tissue, there are methods described in the literature by which their structural, electrophysiological, or metabolic maturity can be increased. Such strategies include the use of nanofibrous mats that allow them to reflect natural conditions in the body more accurately. This offers the hope of obtaining iPSC-CMs with properties more similar to mature cardiomyocytes. Consequently, nanofibers have the potential to be used as structures in 3D cellular models of the heart in studies to develop new cardiac models. However, further research into the use of nanofiber mats and iPSC-CM cultures is still needed. This would allow us to evaluate the use of long-term culture, including the effects on cardiac cell viability and maturation, in order to understand in detail the potential for their use. Understanding the impact of nanofiber properties on the maturation of cardiomyocytes derived from induced pluripotent stem cells (iPSC-CMs) is crucial for the development of cardiac tissue engineering and regenerative medicine, as it can help optimize the conditions for obtaining more mature and functional cardiomyocytes.

The purpose of this study was to investigate the applicability of nanofibrous mats made of polyurethane (PU) and poly(ε-caprolactone) (PCL) with different elasticities as materials for the maturation of iPSC-CMs. Analysis of cell morphology and orientation, as well as the expression levels of selected genes and proteins, was performed to determine the effect of this type of substrate on the maturation of iPSC-CMs after long-term (10-day) culture.

## Results

### Characterization of nanofibrous scaffolds

**Figure 1:**
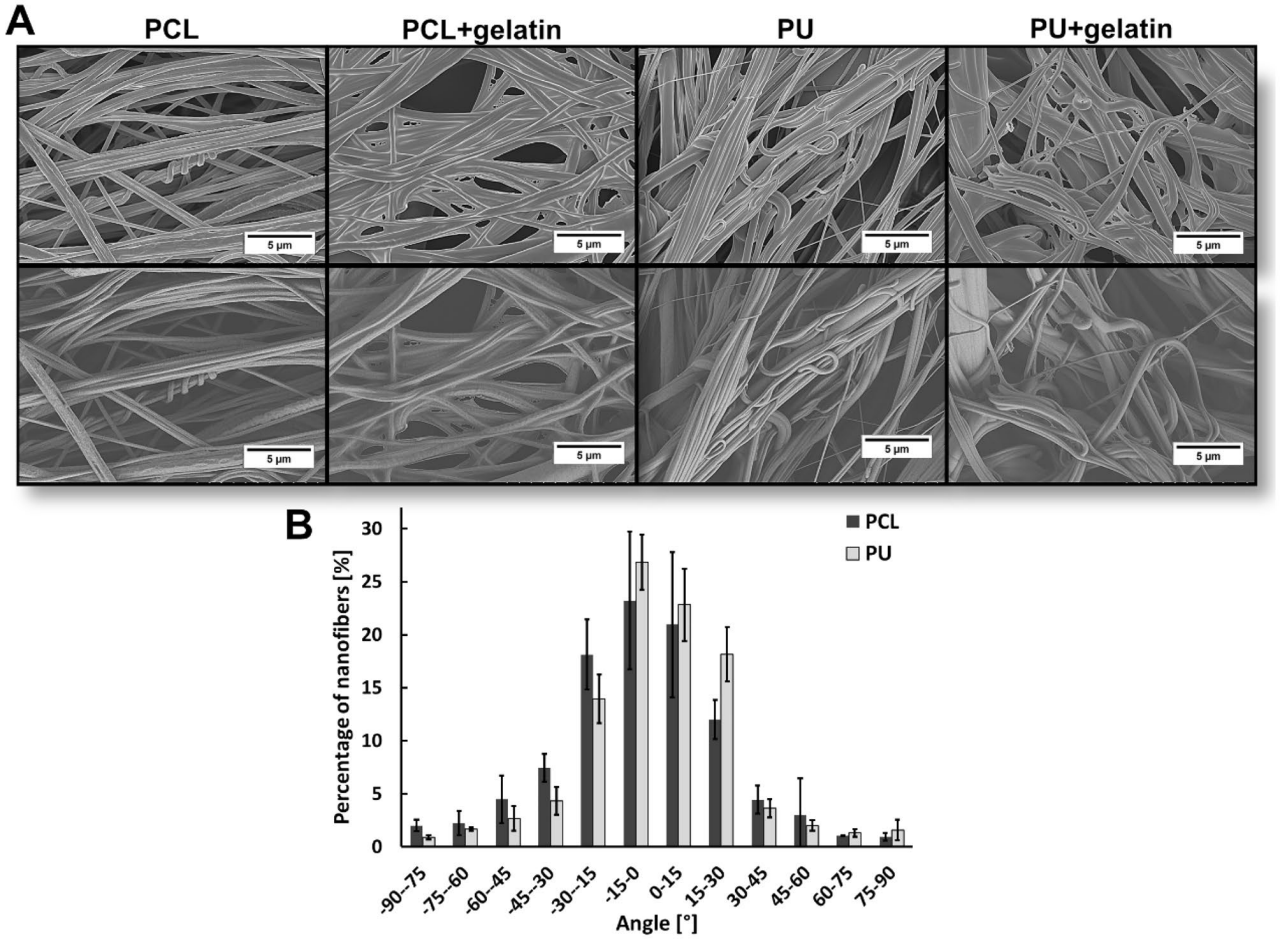
(**A**) SEM images of polycaprolactone (PCL) and polyurethane (PU) nanofibrous mats with or without gelatin coating and (**B**) percentage of nanofibers in relation to the angle of alignment.

The nanofibers used in in vitro cardiac models are most often produced by electrospinning (ES) and solution blow spinning (SBS)^26^. ES is cheap and simple methods however, requires high voltage (up to 60 kV) to produce fibers which results in high polymer consumption therefore, low production efficiency^27,28^. SBS is low-cost, fast, has desirable diameters of fibers, and can obtain parallel arrangements of nanofibers. Moreover, SBS is more efficient than ES in fiber production and nanofibers can have higher porosity and elasticity^26,29,30^. In our study, PCL and PU nanofibrous mats were produced using the solution blow spinning (SBS) technique^18^. Structural properties such as diameter, elasticity, and parallel orientation are shown in Fig. 1 and Table 1. Additionally, we used a gelatin solution to coat nanofibrous mats before seeding iPSC-CMs. Figure 1 shows that gelatin covered the nanofibers. This increases the nanofibers’ biocompatibility, improves cell adhesion, and provides a supportive microenvironment that resembles the natural conditions of cardiac tissue.

**Table 1:** Physical properties of polycaprolactone (PCL) and polyurethane (PU) nanofibers.

	PCL	PU
Diameter [nm]	509 ± 178	452 ± 151
Young’s modulus [MPa]	48.6 ± 3.6*	60.3 ± 8.9*

The tensile strength was measured parallel to the fiber orientation. *p < 0.05- statistically significant differences were determined based on one-way ANOVA. n ≥ 3.

### Characterization of hiPSC-CMs

**Figure 2:**
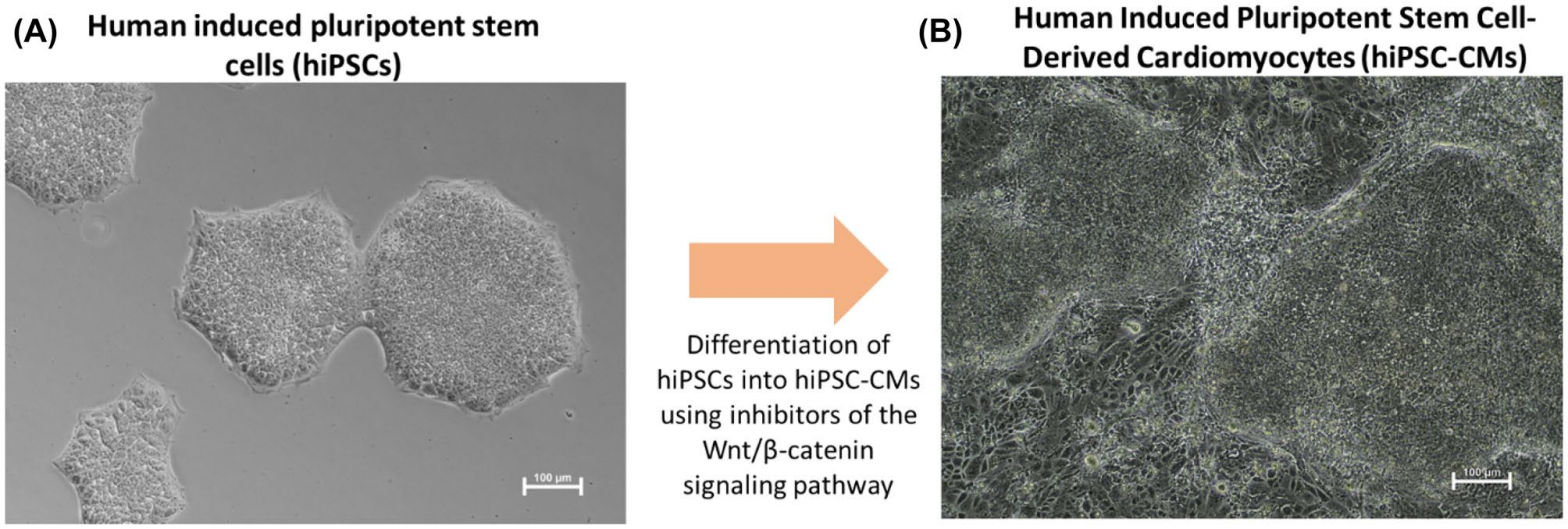
(**A**) iPSCs cultured on PS plate. (**B**) The differentiated hiPSCs (hiPSC-CMs) using inhibitors of the Wnt/β-catenin signaling pathway.

The process of differentiation of hiPSCs into hiPSC-CMs is based on the modulation of the Wnt/β-catenin signaling pathway under the influence of the GSK3 pathway inhibitor (CHIR99021) and the Wnt pathway inhibitor—IWP-2 (Fig. 2). The first spontaneous cell contractions can be observed between 8 and 14 days after the start of differentiation (Movie 1). Additionally, flow cytometry, RT-PCR, and immunofluorescence staining were used to analyze the efficiency of differentiation of hiPSCs into cardiomyocytes (CMs) (Supplementary Material Figs. S1, S2, S3, and S4). The efficiency of the differentiation process was 90.9%, as estimated by the percentage of cTnT-positive cells.

### Determination of the optimal seeding density of iPSC-CMs cells on nanofibrous mats

**Figure 3:**
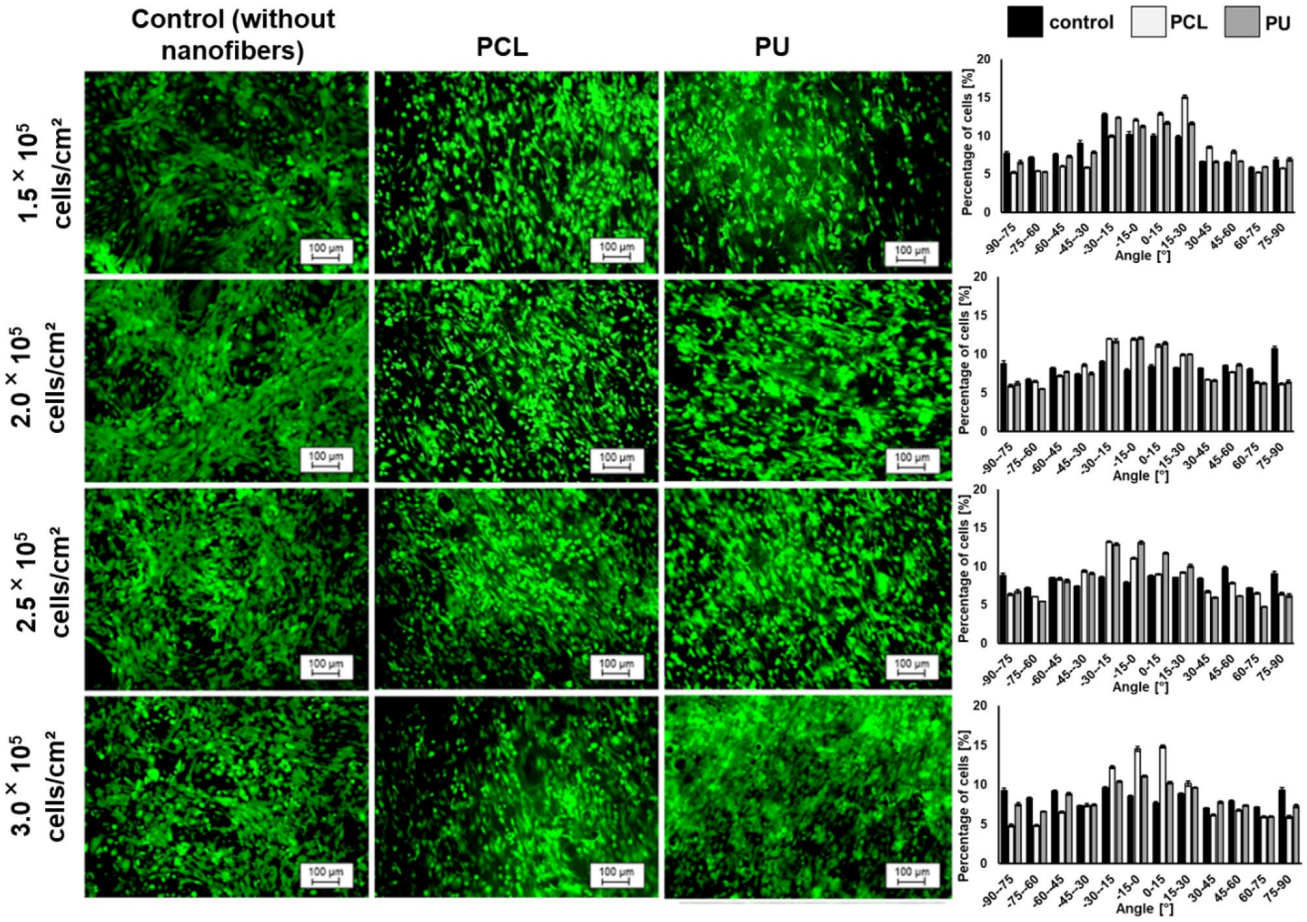
The iPSC-CMs stained with calcein-AM (CAM) (green color) were cultured for 72 h on different nanofibrous mats depending on cell seeding density. A polystyrene plate was used as a control. On the right side are graphs showing the alignment of the cells (for three independent experiments).

To determine the optimal density of iPSC-CMs, the cells with 1.5 × 10^5^ cells/cm^2^, 2 × 10^5^ cells/cm^2^, 2.5 × 10^5^ cells/cm^2^, and 3.0 × 10^5^ cells/cm^2^ were seeded on nanofibrous mats. The iPSC-CMs were initially seeded in a pre-culture medium, optimal for cell passage, then cultured in this medium for 48 h for efficient attachment of the cells to the nanofibers, followed by a 24-h culture period in a standard medium. Three days later, calcein-AM staining was performed. According to Fig. 3 and Table 2 and Fig. S5 (Supplementary Material), high cell viability for cultures on PCL nanofibrous mats, PU nanofibrous mats, and polystyrene plates (control) was noticed. In addition, the cells grown on PCL and PU nanofibrous mats seeded at densities of 2.0 × 10^5^ cells/cm^2^ and 2.5 × 10^5^ cells/cm^2^ showed the most elongated and rod-like shape compared to the control and cultures at other densities. Literature sources show that mature cardiomyocytes have large, anisotropic, and rod-like, elongated shapes (high length/width ratio and less roundness)^10^, while fetal cardiomyocytes are rounder and smaller than adult CMs^31^. iPSC-CMs cultured on nanofibrous mats do not show the significant parallel alignment characteristic of mature cardiomyocytes compared to controls, regardless of seeding density. Instead, seeding density and culturing on nanofibers affect cell elongation. It can be noticed that induced pluripotent stem cell-derived cardiomyocytes grown on PCL nanofibrous mats have the most rod-like shape (4.4 length/width ratio and 45.2% roundness, whereas for PS 2.4 length/width ratio was 63.7% roundness) at densities of 2.5 × 10^5^ cells/cm^2^. In contrast, iPSC-CMs cultured on PU nanofibrous mats have the highest length/width ratio and the lowest roundness (4.7 length/width ratio and 39.6% roundness) for cultures seeded at a 2.0 × 10^5^ cells/cm^2^ density. Based on the above results, 2.0 × 10^5^ cells/cm^2^ seeding density on nanofibrous mats and a PS plate was selected for further research.

**Table 2:** Morphological parameters of iPSC-CMs depend on cell seeding density.

Density [cells/cm^2^]	Nanofibers	Length/width ratio	Roundness [%]
1.5 × 10^5^	Control	2.6	56.3
	PCL	3.3	55.4
	PU	4.2*	47.4
2 × 10^5^	Control	2.7	60.3
	PCL	3.6	47.0
	PU	4.7*	39.6*
2.5 × 10^5^	Control	2.4	63.7
	PCL	4.4*	45.2*
	PU	3.5	52.1
3 × 10^5^	Control	2.1	71.3
	PCL	4.1*	46.8*
	PU	3.4	56.4*

*p < 0.05- statistically significant differences were determined based on one-way ANOVA. n ≥ 3.

### Analysis of the maturation of cardiac cells

**Figure 4:**
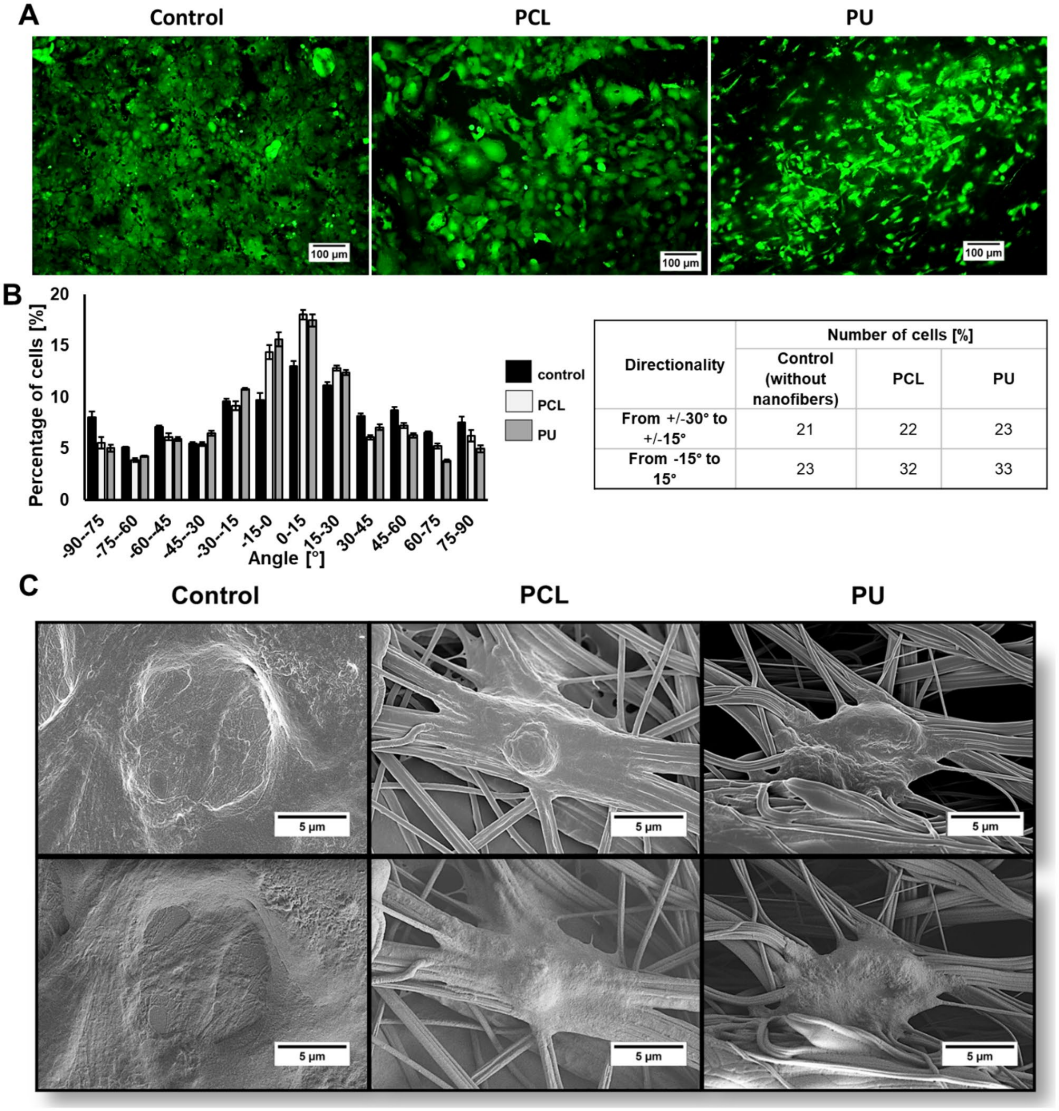
(**A**) iPSC-CMs stained with calcein-AM (CAM) (green color) after 10 days of cell culture on PCL and PU nanofibrous mats. A polystyrene plate was used as a control, scale bars were 100 µm. (**B**) Graphs and tables show the alignment of the iPSC-CMs on the 10th day of culture. The orientation of nanofibers was assumed to be 0°, (**C**) SEM images showing iPSC-CMs on polystyrene plate (Control) and nanofibers (PCL and PU) after 10 days, scale bars 5 µm.

To investigate the maturation of iPSC-derived cardiomyocytes on nanofibrous mats, we analyzed cell morphology, anisotropy, genes and proteins specific to mature cardiomyocytes after 10 days of culture. Calcein-AM was used to stain the cells, and fluorescence microscope images were taken. Based on them, the morphology and orientation of cells growing on PS plate, PCL, and PU nanofibrous mats were analyzed (Fig. 4 and Table 3). Image 4C shows that cells cultured on PS are flattened and grow in a monolayer, while cells growing on nanofibers are entwined by nanofibers distributed at different levels of the scaffold and show three-dimensional growth. Due to the three-dimensional growth of the cells, it can be a problem to take a picture of all the living cells stained with calcein-AM at different levels of culture (Fig. 4A). It can be observed that a round shape of cells characterizes the control culture (without nanofibers) and is randomly oriented. In contrast, iPSC-CMs cultures grown on both types of nanofibers have a more elongated and rod-like shape than the control (for cultures grown on PCL nanofibers were 2.7 length/width ratio and 49.3% roundness, for PU nanofibrous mats were 3.2 length/width ratio and 46.9% roundness, whereas for the control length/width ratio was 1.5 and roundness was 63.6%). In addition, the orientation of the cells is parallel to each other, unlike those cultured on a PS plate. Indeed, all types of nanofibers led to an increase in the number of parallel arrangements of iPSC-CMs. For the control, this was observed in 23% of the cells with an alignment of -15° to 15°, while culture conducted on PCL nanofibers led to an increase to 32% and on PU to 33%. Comparing short (3-day) and long (10-day) cultures of iPSC-CMs, it can be seen that the cells cultured on nanofibrous mats have a similar elongated and rod-like shape. In contrast, cells cultured on PS after 10 days show a rounder shape (less length/width ratio and roundness) than cultures after 3 days.

**Table 3:** Morphological parameters of iPSC-CMs after 10 days of culture.

Surface	Length [μm]	Width [μm]	Length/Width ratio	Roundness [%]
Control	25.1	16.5	1.5	63.6
PCL	32.9	12.1	2.7*	49.3*
PU	45.4	14.4	3.2*	46.9*

*p < 0.05- statistically significant differences were determined based on one-way ANOVA. n ≥ 3.

**Figure 5:**
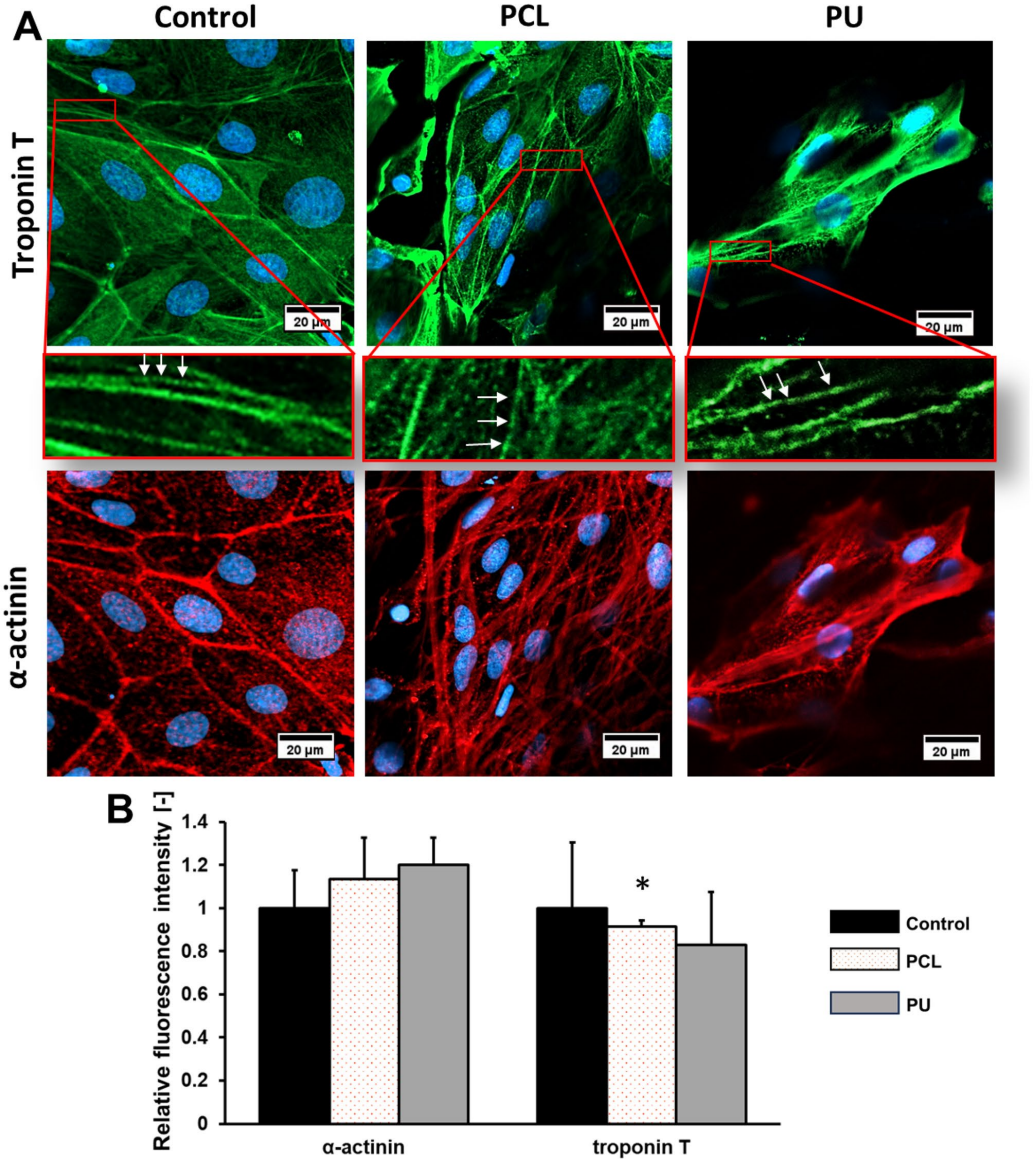
(**A**) Immunofluorescence staining of α-actinin (Alexa Fluor 594, red fluorescence), troponin T (Alexa Fluor 488, green fluorescence) with sarcomere structure, and nucleus (Hoechst 33,342, blue fluorescence) of iPSC-CM, and (**B**) relative fluorescence intensity were performed after 10 days of culture on a polystyrene plate (control) and PCL and PU nanofibrous mats. Scale bar 20 µm. *p < 0.05 – statistically significant differences were determined by comparison with the cells cultured on a polystyrene plate (control) (the Student's *t*-test). n ≥ 3.

**Figure 6:**
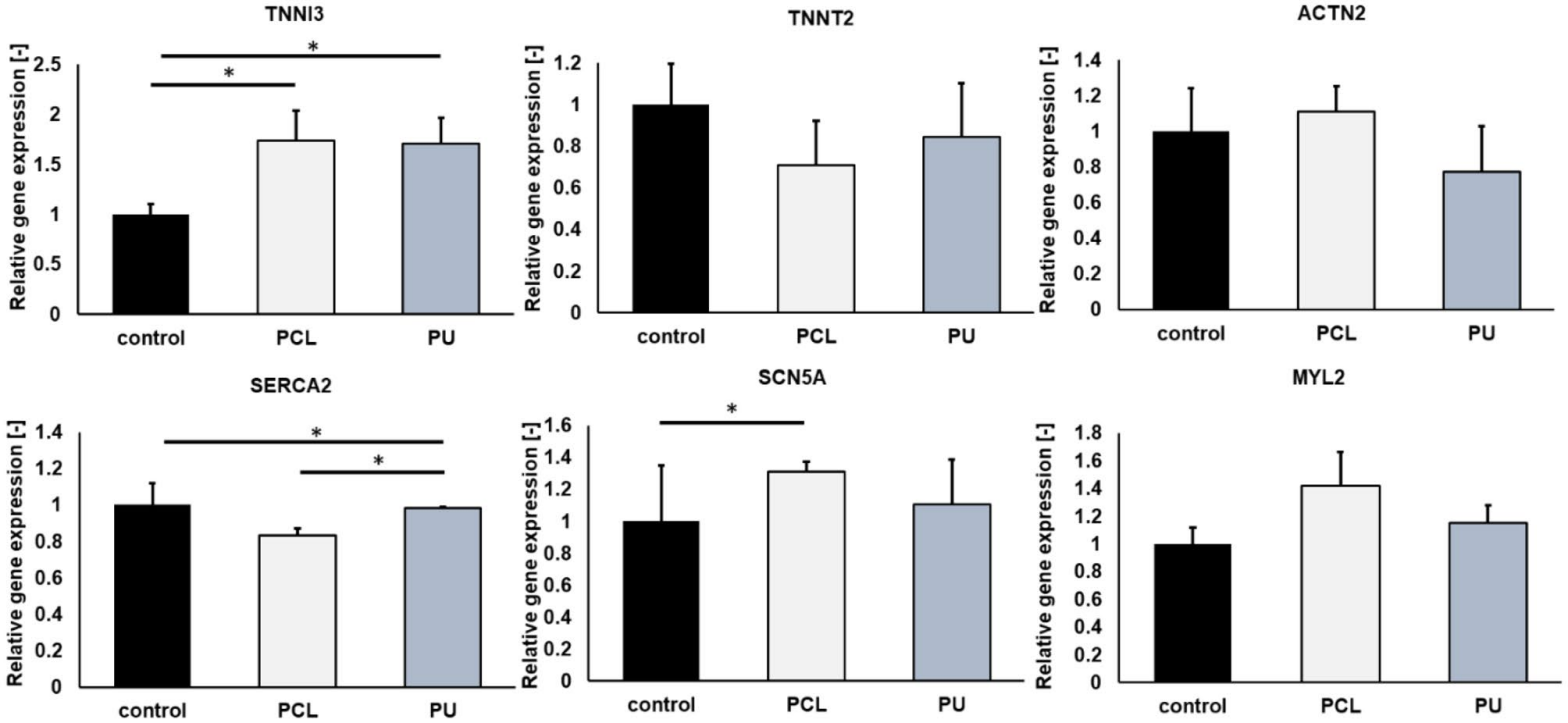
Quantitative qRT-PCR analysis of gene expression from cardiac cells cultured on polystyrene plate, PCL and PU nanofiber mats for 10 days. Gene expression of troponin T (TNNT2), troponin I (TNNI3), cardiac α-actinin (ACTN2), calcium ATPase type 2 (SERCA2), sodium channel protein type 5 subunit alpha (SCN5A) and myosin regulatory light chain 2 (MYL2) for iPSC-derived cardiomyocytes. *p < 0.05 – statistically significant differences were determined by comparison with the cells cultured on a polystyrene plate (control) and nanofibrous mats (one-way ANOVA followed by Tukey's post hoc test). n = 3.

After the maturation of iPSC-CMs on nanofibrous mats and PS for 10 days, cardiac-specific genes and proteins were examined using immunofluorescence staining and RT-PCR techniques. The presence of cardiomyocyte-specific proteins, such as α-actinin (labeled in red) and troponin T (labeled in green), were compared (Fig. 5). Additionally, the level of gene expression, such as *TNNT2* (troponin T), *TNNI3* (troponin I), *SERCA2* (calcium-ATPase type 2), *ACTN2* (cardiac α-actinin), *MYL2* (myosin regulatory light chain 2), and *SCN5A* (sodium voltage-gated channel alpha subunit 5) were determined (Fig. 6). Based on the images and graphs in Fig. 5, the expression of cardiomyocyte marker sarcomeric α-actinin is higher for cultures iPSC-CMs grown on both types of nanofibers in comparison than for control in 10th day culture. For cultures grown on PCL and PU nanofibrous mats, it was 1.1-fold and 1.2-fold higher than the control, respectively. Moreover, the expression of troponin T protein in human iPSC-derived cardiomyocytes was determined. In iPSC-CMs cultures grown on nanofibers, the expression cTnT decreased (for PCL it is 0.9-fold, while for PU is 0.8-fold). In our study, we used antibodies, such as anti-troponin T, specific for cTnT/*TNNT2*, the expression of which is higher in immature human cardiomyocytes. The decrease in troponin T expression may be related to the maturation of human cardiomyocytes on nanofibers, which in turn is associated with a switch of cTnT1 and cTnT2 isoforms in favor of cTnT3 and cTnT4 isoforms in maturing cardiac cells. According to the literature, *TNNT2* is an isoform of troponin T, which is higher in fetal CMs, than in adults^32^. Also, this correlation can be noticed in Fig. 6 with quantitative analysis of *TNNT2* gene expression. *TNNT2* is an isoform predominantly in fetal human cardiomyocytes, and for iPSC-CMs the decrease in *TNNT2* expression was 0.7-fold for PCL nanofibers and 0.8-fold for PU scaffolds compared to the PS cultures. In contrast, *TNNI3* is the predominant isoform of the troponin I gene (*TNNI*) in adult CMs, for iPSC-CMs cultured on PCL and PU nanofibers, the increase statistically significant in *TNNI3* expression and was 1.7-fold compared to control.

In addition, an increase in the expression levels of *SCN5A, MYL2,* and *ACTN2* genes was observed for cultures on PCL nanofibers, compared to cultures on PS. An increase in *SCN5A* and *MYL2* gene expression was also observed for cultures on PU nanofibers. In both polycaprolactone and polyurethane substrates, a significant decrease in the expression of *SERCA2*, which encodes an ATPase responsible for calcium ion transport, was observed.

## Discussion

In cardiac tissue engineering, an increasing number of literature reports have indicated the potential of using human iPSCs and the possibility of differentiating them into cardiomyocytes. The derivation of human induced pluripotent stem cells does not involve ethical concerns as in the case of embryonic stem cells, which can also differentiate into human cardiomyocytes. This gives them a significant advantage in the context of being used to develop in vitro cell models and later for therapeutic purposes. In addition, they can be obtained from fibroblasts present in the skin, eliminating the need for invasive procedures^33^. iPSCs can be differentiated into cardiomyocytes by stimulating with appropriate factors, which model the canonical Wnt signaling pathway. iPSC-CMs are most often differentiated in a 2D model (monolayer) to result in immature cells compared to adult cardiomyocytes in vivo^34^. The monolayer culture method does not include 3D structure, which considers the numerous cell–cell interactions present in vivo. For this reason, 3D models have become increasingly approved because of more appropriate imitations of the structure and function of native cardiac tissue. For 3D models of stem cell-derived cardiomyocytes, we can include organoids or cultured iPSC-CMs on nanofibrous mats.

An organoid is a multicellular 3D structure composed of cell types specific to a particular organ or tissue capable of self-organization in vitro. Organoids formed from stem cells adopt a 3D structure mimicking native tissue and can differentiate into cardiomyocytes and other cardiac cells, such as cardiac fibroblasts, endothelial and epicardial cells^35^. Also, it is possible to differentiate iPSCs into iPSC-CMs and then culture them into 3D structures. One type of scaffold used in in vitro heart models is 3D nanofibrous scaffolds, which can structurally and functionally mimic the extracellular matrix of cardiac tissue. It is important to create a structure that mimics the native extracellular matrix (ECM) to study in vitro cardiac tissue models, including cardiac cell function, cell–cell and cell-structure communication, cell proliferation, and differentiation. Nanofibers are used to culture iPSC-CM cells, which are characterized by immaturity in relation to in vivo cardiomyocytes. Nanofibers can help enhance the characteristics of mature cardiomyocytes in cardiac tissue and enable their use in 3D cardiac models^14,25,36^.

The culture of iPSC-CMs and observation of maturation of these cells on nanofibrous mats has been reported in several works^14,36–40^. The most commonly used nanofibers in research are those made from biodegradable polymers such as PCL, PLGA, and PU^14,25,38–40^, therefore in our study, we have selected two types of polymers most commonly used in research. However, there is a lack of comparison on whether nanofibers' physicochemical properties (polymer type, elasticity) affect the functioning of iPSC-CMs. In the present study, nanofibrous mats made of PCL and PU with similar diameters and different elasticities (PCL scaffolds had modulus of 48.6 ± 3.6 MPa, and PU scaffolds had modulus of 60.3 ± 8.9 MPa) were used to culture iPSC-CM cells to investigate whether they affect the function of cultured cardiomyocytes. According to the current literature, materials with Young's modulus ranging from 20 kPa to 92 MPa were used to produce nanofibers for cardiac cell culture and were biocompatible with them^41,42^ and a wide range of nanofibers used depends on the manufacturing method and type of material^43^. The research cited here indicates that iPSC-CMs cultured on PCL and PU nanofibrous scaffolds show similar, more mature morphology and expression of cardiomyocyte-specific proteins and genes compared to polystyrene plate cultures, even though nanofibrous mats differ in elasticity and polymer type. Comparing the results in this article with literature sources in which nanofibers with parallel orientation were used to mature iPSC-CMs, it was noted that it affects elongation and parallel alignment of cardiomyocytes. Moreover, nanofibers affect the stimulation of protein and gene expression, which change similarly during cardiomyocyte maturation in vivo. However, there is a lack of information in the literature on the effects of biomaterial composition, diameter and elasticity of nanofibers on cardiomyocyte function^14,25,38–40^, which was investigated in this study.

In many literature sources, cardiomyocytes differentiated from induced stem cells are cultured on different types of nanofibrous scaffolds, but there is a lack of studies evaluating the effect of seeding density on cell viability and morphology, and thus on their maturation^14,37,38,40^. Additionally, there are reports in which iPSC-CMs seeded at a much higher density (10^6^–4 × 10^7 ^cells/cm^2^)^44–46^ were used for studies. However, the cells were utilized as patches for transplantation and in vivo studies. Moreover, more cells are required due to the high cell death rate after transplantation. Our research focused on cell function on selected nanofibrous mats, and it was essential to use lower cell density. A much lower cell density is crucial for adequately evaluating images after staining. This density was sufficient to receive sarcomeres in the cells. It can also be seen that the use of cells seeded at lower densities showed more mature morphology and physiology cultured on nanofibrous scaffolds^14^. Our study was carried out with four different densities of seeding (1.5 × 10^5^ cells/cm^2^, 2 × 10^5^ cells/cm^2^, 2.5 × 10^5^ cells/cm^2^ and 3.0 × 10^5^ cells/cm^2^). Analysis of the morphology and orientation of cells depending on their seeding density allowed us to choose the most optimal initial cell density of iPSC-CMs. The study showed that for PU nanofibrous mats, it is 2 × 10^5^ cells/cm^2^, while for PCL it is 2.5 × 10^5^ cells/cm^2^ because cells in these densities are the most morphologically similar to mature cardiomyocytes. Moreover, at these densities, the cells have the highest length-to-width ratio and less roundness than controls. However, an analysis of the orientation of iPSC-CMs showed no significant effect of initial cell density on cell alignment.

Further studies were performed ten days after the start of culture, showing that the use of nanofibrous mats affects the maturation of iPSC-CM cells. The averaged morphology results in Table 3 show that culturing cells on nanofibrous mats increased the length-to-width ratio of iPSC-CMs cells compared to controls. This resulted in more elongated cells characterized by less roundness, and their morphology is closer to that of mature cardiomyocytes^47^. For both types of nanofibrous mats, a similar effect on cell maturation in terms of morphology was observed to compare with the control. Additionally, the use of PCL and PU nanofibers led to an increase in the number of iPSC-CMs with parallel orientation. The parallel arrangement of cells can influence the maturation of iPSC-CMs which has been confirmed in other scientific studies^48,49^. After comparing cell morphology and alignment, it is necessary to evaluate the expression of specific cardiac genes and proteins, which increases or decreases with cardiac cell maturation^50^. The analysis showed that iPSC-CMs cultured on PCL nanofibrous mats have significant levels of structural genes and proteins such as *TNNI3* and *MYL2* genes, similar to the study by Chun et al.^51^ research, as well as increased expression of *ACTN2* gene and a higher level of α-actinin protein than iPSC-CMs cultured on PS plate. In addition, there was a decrease in *TNNT2* gene and cTnT2 protein expression, which may also indicate the maturation of iPSC-CMs grown on nanofibers^32^. Cultures grown on PU nanofibrous mats contributed to a slight increase in the expression of *TNNI3,* and *MYL2* genes. Additionally, iPSC-CMs grown on PU nanofibers have the highest level of expression of α-actinin and the lowest expression of cTnT2 proteins compared with cells cultured on PCL nanofibers and PS. In this study, we observed altered expression levels of *SCN5A* and *SERCA2* genes, which are involved in electrophysiological processes of cardiac tissue in vivo^34^^–^^36^, was noticed. The *SCN5A* gene encodes the Nav 1.5 protein, which builds the voltage-gated sodium channel subunit. The channel is responsible for the rapid increase in action potential and plays a key role in cardiomyocyte contraction^52^. In vivo, *SCN5A* gene expression increases with cardiac tissue development^10^. In the case of *SERCA2*, which encodes an ATPase responsible for calcium ion transport^53^, a decrease in the expression level of this gene has been observed, but to our knowledge, there is no information as to why the 3D scaffold can induce such a response in iPSC-CMs.

Previous study has noted that depending on the biomaterial, different maturation mechanisms are induced in cardiac cells isolated from the ventricle and atrium^54^. According to the literature, ventricle cells require more elastic biomaterials like PCL, while atrial cardiomyocytes require more stiffness materials such as PU. Moreover, atrial and ventricular cardiomyocytes differ electrophysiologically (including different action potentials), structurally, and functionally^55^. Therefore, biomaterials that distinct in functional groups may affect cardiomyocyte maturation differently^55^. iPSC-CMs have been differentiated from iPSC, and their population includes both ventricular and atrial cardiomyocytes^56^; thus, PCL and PU nanofibers stimulate the maturation of iPSC-CMs; however, they may induce different mechanisms.

Both types of nanofibrous materials used during the study proved to be suitable substrates for iPSC-CMs. Moreover, both types of nanofibers led to an increase in the number of cells with parallel orientation. Cells cultured on PU nanofibrous material had the most elongated shape and the least roundness, and expressed proteins that are more characteristic of cardiomyocyte maturation. In contrast, the culture on PCL nanofibers resulted in the greatest changes in the expression levels of the studied genes, indicating an increase in cell maturity. Both types of nanofibrous materials stimulated myocardial cell maturation, but probably through different mechanisms.

## Conclusion

In this study, long-term cultures of human induced pluripotent stem cell-derived cardiomyocytes (iPSC-CMs) were grown on nanofibrous mats made of polycaprolactone (PCL) and polyurethane (PU). The study was conducted on nanofibers differing in the polymer used, and hence, their elasticity. Despite the physicochemical differences between the nanofibrous scaffolds, both types of nanofibrous materials are suitable substrates for increasing the maturity of iPSC-CM cells. Based on these results, it can be concluded that human cardiac cells cultured on nanofibrous mats lead to an increase in cell maturity in terms of their orientation, morphology, genes, and protein expression levels compared to cells grown on polystyrene plates. These studies can support research on understanding and explaining the mechanisms leading to cellular maturity in the heart and the selection of nanofibers that will effectively help the maturation of cardiomyocytes.

## Materials and methods

### Fabrication of nanofibrous materials

Poly(ε-caprolactone) (PCL, Sigma Aldrich Mn = 80,000) and polyurethane (PU, ChronoFlex C75D, AdvanSource Biomaterials) nanofibrous mats were prepared using solution blow spinning (SBS). PCL was dissolved in 2–2-2-trifluoroethanol (> 99%, ABCR) at a concentration of 10% (w/w). PU solutions in 1,1,1,3,3,3-hexafluoroisopropanol (> 99%, ABCR) were prepared at a concentration of 5.5% (w/w). PCL nanofibers had an average fiber diameter of 509 ± 178 nm and elasticity (Young's modulus) of 48.6 ± 3.6 MPa. PU scaffolds had an average fiber diameter of 452 ± 151 nm and elasticity (Young's modulus) of 60.3 ± 8.9 MPa. A high porosity of the obtained biomaterials was observed. It was 83 ± 2% and 82 ± 2% for PCL and PU, respectively). Our previous research described the fabrication and characterization of nanofiber's morphology in detail^57^.

### Culture of human induced pluripotent stem cells (hiPSCs)

Human induced pluripotent stem cells (hiPSCs, IIMCBi001-A (ELE10) line, the Laboratory of Molecular and Cellular Neurobiology of the International Institute of Molecular and Cell Biology in Warsaw) were obtained by reprogramming dermal fibroblasts by using a lentiviral vector delivering *OCT4, SOX2, KLF4*, and *C-MYC.* The dermal fibroblasts were isolated from a skin biopsy of a 10-year-old healthy girl, Caucasian origin. The protocol for obtaining hiPSCs and their characteristics are presented in the article by Liszewska et al.^58^. hiPSCs were grown in Essential 8™ Medium (E8, Thermo Fisher Scientific) supplemented with 5 μM ROCK inhibitor (Tocris) by first 24 h culture, on Matrigel (Corning) coated 6-well plates (Sarsedt). The cultures were maintained in a humidified incubator (37 °C, 5% CO_2_, HERA-cell 150, Thermo Scientific) to obtain 90% confluence, then washed with Dulbecco’s Phosphate Buffered Saline (DPBS, ATCC) and passaged using Versen (Thermo Fisher Scientific).

### Differentiation of hiPSC into hiPSC-CMs

Differentiation of hiPSCs into hiPSC-CMs was performed according to the GiWi protocol described by Lian et al.^8^. The process is based on the modulation of the pathway with the GsK3 inhibitor and Wnt inhibitor. The iPSCs were cultured in an E8 medium until they reached 90% confluence. Next, the old medium is aspirated and added RPMI (Thermo Fisher Scientific) medium with B-27 without insulin (Thermo Fisher Scientific) and supplemented with 12 μM (final concentration) CHIR99021 (Tocris). After 24 h, the medium was changed to RPMI/B-27 without insulin. On 3rd day of the differentiation (72 h after the addition of CHIR99021), half the volume of the medium was removed, and the other half the volume of RPMI/B-27 medium without insulin supplemented with 5 μM (final concentration) IWP-2 (Tocris) was added. On the 5th day of the differentiation, the medium was changed to RPMI/B-27 without insulin. After another 2 days, the medium was changed to RPMI/B-27 with insulin and replaced every 3 days. Following the GiWi protocol, it is possible to obtain cells that are characterized by spontaneous beating approximately 14 days after the start of hiPSCs differentiation. The cultures were maintained in a humidified incubator (37 °C, 5% CO_2_).

### hiPSC-CMs culture on nanofibrous mats

The nanofibrous mats were placed in a 24-well plate, sterilized with 70% EtOH (POCH) solution for 30 min, and dried. The surface of nanofibrous materials was modified with oxygen plasma (Diener) and covered with 0.1% (wt/vol) gelatin solution (Sigma-Aldrich) to improve their hydrophilic properties. 24 h later, the protein solutions were removed. Next, the nanofibrous mats were washed with a culture medium, and the cells were seeded on nanofibrous mats. For this purpose, the human induced pluripotent stem cell-derived cardiomyocytes were washed with DPBS and detached by TrypLE (Thermo Fisher Scientific). Cells are resuspended in RPMI medium with 20% v/v fetal bovine serum (FBS, Gibco), 1% v/v 100 mM Penicillin–Streptomycin (Sigma-Aldrich) and 5 μM ROCK inhibitor. hiPSC-CMs were seeded on the prepared nanofibrous mats and polystyrene plate (PS as a control – was also covered with a gelatin solution and incubated for 24 h) with density 1.5 × 10^5^, 2 × 10^5^, 2.5 × 10^5^ and, 3 × 10^5^ cells/cm^2^. After 48 h, the medium was replaced by RPMI/B-27 with insulin and exchanged every 3 days.

### Calcein-AM assay

Cell morphology and arrangement were determined by microscopic observations (Nikon Eclipse Ts2-FL) after being stained with Calcein-AM (CAM, Sigma-Aldrich) at 0.5 µg/ml final concentration in RPMI medium. Living cells were observed on the 3rd and 10th day of cultures. Observation and photos were carried out using a fluorescence microscope with a filter in the 470–510 nm wavelength range. Then, these images were analyzed using the ImageJ program.

### Immunofluorescence staining of hiPSC-CMs

iPSC-CMs were fixed using 4% paraformaldehyde for 10 min at room temperature (RT). Next, cultures were permeabilized by 0.5% Triton X-100 (Sigma-Aldrich) in DPBS and blocked by 2.4% bovine serum albumin (BSA, Thermo Fisher Scientific) solution in DPBS (50 min, RT). Cells were incubated with primary antibody at 4 C overnight: rabbit monoclonal anti-troponin T (1:100) (Abcam), mouse monoclonal anti-α-actinin (1:100) (Sigma-Aldrich), anti-NKX2.5 (1:100) (Santa Cruz Biotechnology), anti-GATA4 (1:100) (Santa Cruz Biotechnology), anti-ANP (1:100) (Santa Cruz Biotechnology) and then with secondary antibody: goat anti-rabbit Alexa Fluor 488 (1:200) (Thermo Fisher Scientific) and goat anti-mouse Alexa Fluor 594 (1:200) (Thermo Fisher Scientific), donkey anti-mouse Alexa Fluor 488 (1:200) (Thermo Fisher Scientific), goat anti-mouse Alexa Fluor 568 (1:200) (Thermo Fisher Scientific) for 1 h. All antibodies were diluted in a 2.4% BSA. Nuclei were stained with Hoechst 33,342 (10 μg/ml in PBS) (Thermo Fisher Scientific) or DAPI (Thermo Fisher Scientific) (1 μg/ml in PBS) for 5 min. F-actin was stained using the Actin Green 488 Ready Probes reagent (Thermo Fisher Scientific). The stained cells were observed and evaluated under the Olympus BX41 fluorescence microscope, Zeiss Axio Observer 7 + LSM 900 confocal microscope.

### Flow cytometry analysis

Flow cytometry analysis was performed to estimate the efficiency of the differentiation according to the protocol^8^. Briefly, cells from a six-well plate were harvested and labeled with primary anti-troponin T (1:100) (Abcam) or mouse monoclonal anti-α-actinin (1:100) (Sigma-Aldrich) antibodies as well as the appropriate secondary antibodies (donkey anti-mouse Alexa Fluor 488 (1:200) (Thermo Fisher Scientific), goat anti-rabbit Alexa Fluor 488 (1:200) (Thermo Fisher Scientific). The relevant isotypes served as negative controls. Cells were washed three times with FACS buffer, resuspended in 0.5 mL PBS, and loaded into the flow cytometer (FACSCalibur, BD Biosciences). Data were analyzed by using FlowJo software (Tree Star, Ashland, USA).

### Scanning electron microscopy analysis

iPSC-CMs were washed with DPBS three times. Next, the cells were fixed with 4% paraformaldehyde and incubated for 30 min. Then, iPSC-CMs were washed and stored in DPBS for 24 h at 4 °C. After this, samples were dehydrated using the increasing ethanol gradient (5%, 25%, 50%, 75%, and 100%, respectively, each for 15 min). Dehydrated iPSC-CMs were covered with a 20 nm layer of gold–palladium (Quorum Q150 TS, Quorum Technologies) and were evaluated with a Scanning Electron Microscope (SEM, SU 8230, Hitachi High-Technologies Corporation).

### Gene expression analysis

RNA was isolated according to RNeasy Mini Kit (Qiagen) protocol and then reverse transcribed into cDNA using RevertAid H Minus First Strand cDNA Synthesis Kit (Thermo Fisher Scientific). Real-time reverse transcription-quantitative polymerase chain reaction (RT-PCR) was performed using SsoAdvanced Universal SYBR Green Supermix (Bio-Rad) on a CFX Connect Real-Time PCR System (Bio-Rad). Gene expression was normalized to *GAPDH* as a housekeeping gene and calculated using the ΔΔCT method. The sequences of the primers used to analyze the expression of human cardiac-specific genes are shown in the Supplementary Material (Table S1).

### Statistical analysis

Statistical significance was evaluated as the mean ± standard deviation (SD) by Student's t-tests or ANOVA for a minimum of three independent experiments using the OriginPro 8 software. Values of *p* < 0.05 were considered statistically significant and marked with an asterisk.

### Supplementary Information

Supplementary Information.

Supplementary Movie 1.

## Data Availability

The data sets used and/or analyzed during the current study are available from the corresponding author on reasonable request.
