# Supplementary material for: Maturation of human cardiomyocytes derived from induced pluripotent stem cells (iPSC-CMs) on polycaprolactone and polyurethane nanofibrous mats
Source: Sci Rep. 2024 Jun 5;14:12975. doi: 10.1038/s41598-024-63905-z (PMC11153585; doi:10.1038/s41598-024-63905-z)
Supplement: Supplementary file 1 — Supplementary Information. [file 41598_2024_63905_MOESM1_ESM.docx]

Supplementary Material

**Maturation of human cardiomyocytes derived from induced pluripotent stem cells (iPSC-CMs) on polycaprolactone and polyurethane nanofibrous mats**

**Zuzanna Iwoń^a^, Ewelina Krogulec^b^, Inez Tarnowska^a^, Iwona Łopianiak^c^, Michał Wojasiński^c^, Agnieszka Dobrzyń^b^, Elżbieta Jastrzębska^a,d^**

*^a^ Chair of Medical Biotechnology, Faculty of Chemistry, Warsaw University of Technology, Warsaw, Poland*

*^b^ Laboratory of Cell Signaling and Metabolic Disorders, Nencki Institute of Experimental Biology PAS, Warsaw, Poland*

*^c^ Department of Biotechnology and Bioprocess Engineering, Faculty of Chemical and Process Engineering, Warsaw University of Technology, Warsaw, Poland*

*^d^ Centre for Advanced Materials and Technologies, CEZAMAT Warsaw University of Technology, Warsaw, Poland*

** Corresponding author elzbieta.jastrzebska@pw.edu.pl Noakowskiego 3, 00-664 Warsaw, Poland*

**A real-time reverse transcription-quantitative polymerase chain reaction**

Specific cardiac genes whose expression changes with iPSC-CMs maturation were selected for study (Table S1).

Table S 1 The sequence of primer used.

| Gene | Forward Primer | Reverse Primer |
| --- | --- | --- |
| Human, TNNT2 | TTCACCAAAGATCTGCTCCTCGCT | TTATTACTGGTGTGGAGTGGGTGTGG |
| Human, TNNI3 | CGTGTGGACAAGGTGGATGAAG | GCCGCTTAAACTTGCCTCGAAG |
| Human, ACTN2 | GTGAACACCCCTAAACCCGA | AGGGGGATCCTTCCACAGAG |
| Human, MYL2 | CGGAGAGGTTTTCCAAGGAGGA | CTCTTCTCCGTGGGTGATGATG |
| Human, SCN5A | AGGTCGGAAACCTGGTAAGG | TCCTTACCCATGAAGGCTGTG |
| Human, SERCA2 | GGACTTTGAAGGCGTGGATTGTG | CTCAGCAAGGACTGGTTTTCGG |
| Human, GATA-4 | TAGACCGTGGGTTTTGCATTG | CATCCAGGTACATGGCAAACAG |
| Human, NKX2.5 | ACCCTGAGTCCCCTGGATTT | TCACTCATTGCACGCTGCAT |
| Human, cTNNI | CCAACTACCGCGCTTATGC | CTCGCTCCAGCTCTTGCTTT |
| Human, GAPDH | GTGGACCTGACCTGCCGTCT | GGAGGAGTGGGTGTCGCTGT |

**iPSC-CMs analysis after differentiation**


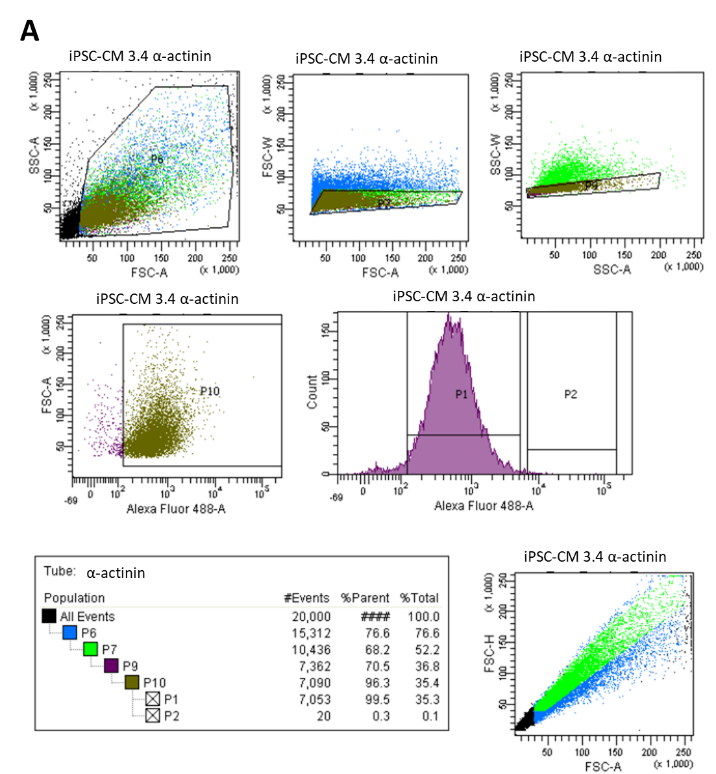

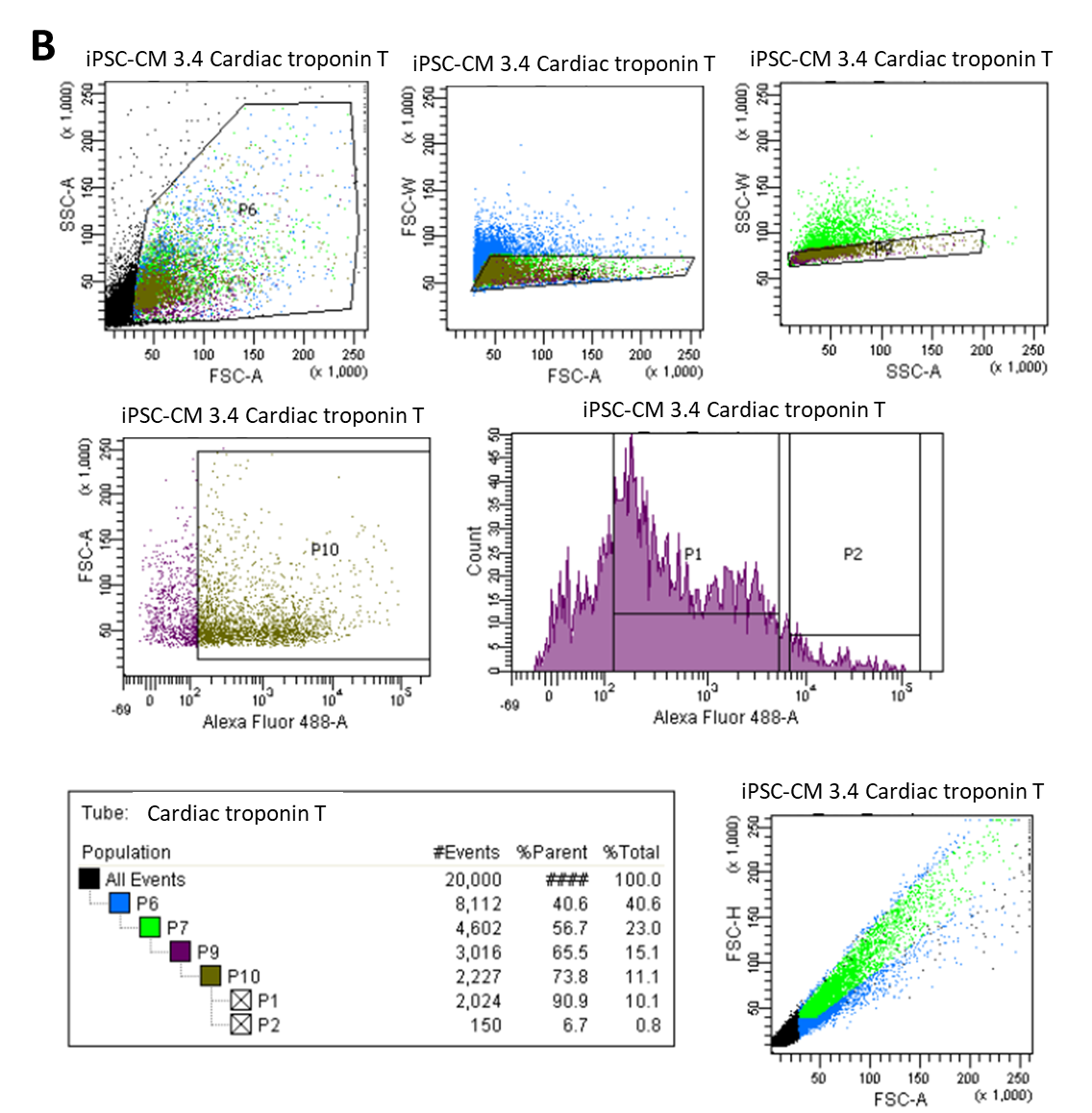


Figure S 1 (A) Percentage of cardiac troponin T - positive cardiomyocytes present at day 15 after differentiation via GiWi protocol with isotype data. (B) Percentage of α-actinin - positive cardiomyocytes present at day 15 after differentiation via GiWi protocol with isotype data. The percentage of positive cells was calculated by flow cytometric analysis of at least 20,000 total cells.


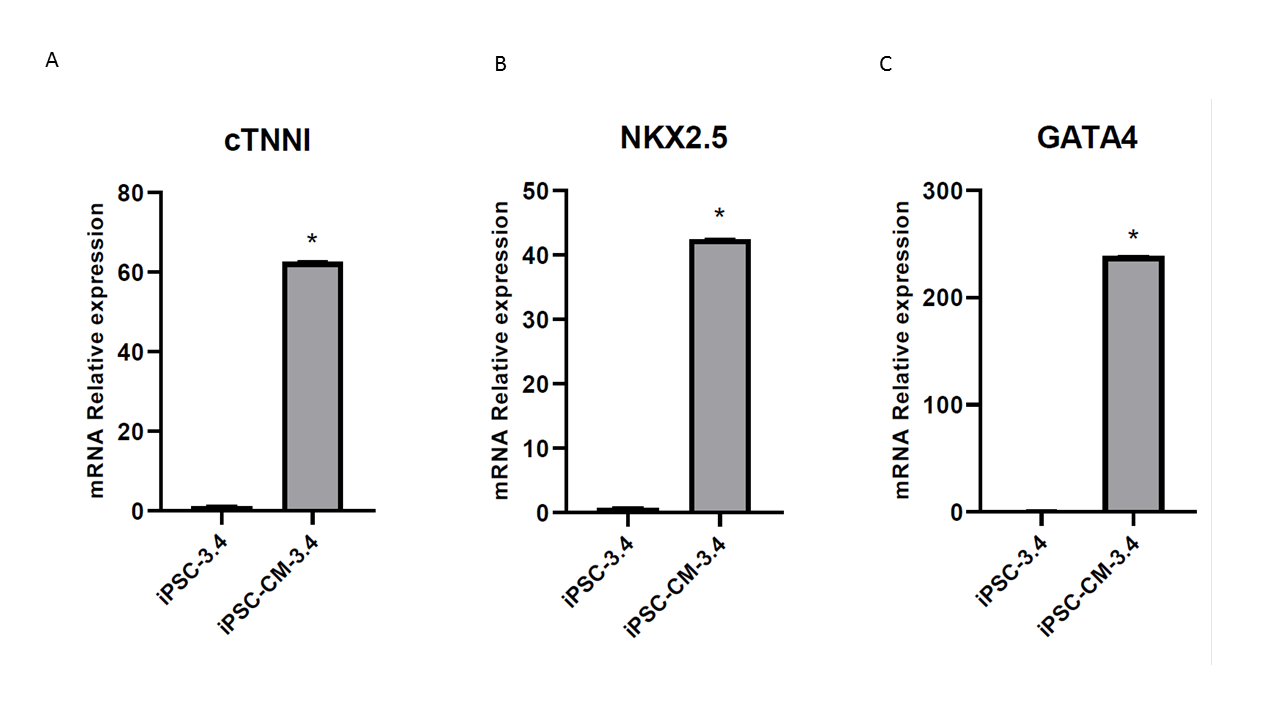


Figure S 2 Cardiomyocyte-specific gene expression present at day 20 after cell differentiation. Results of qRT-PCR analysis of cardiac troponin I (A) and cardiac-related transcription factors NKX2.5 (B) and GATA4 (C). * - p<0.05 – statistically significant differences were determined by Student's t-test. n=3. Error bars show means ± s.d.


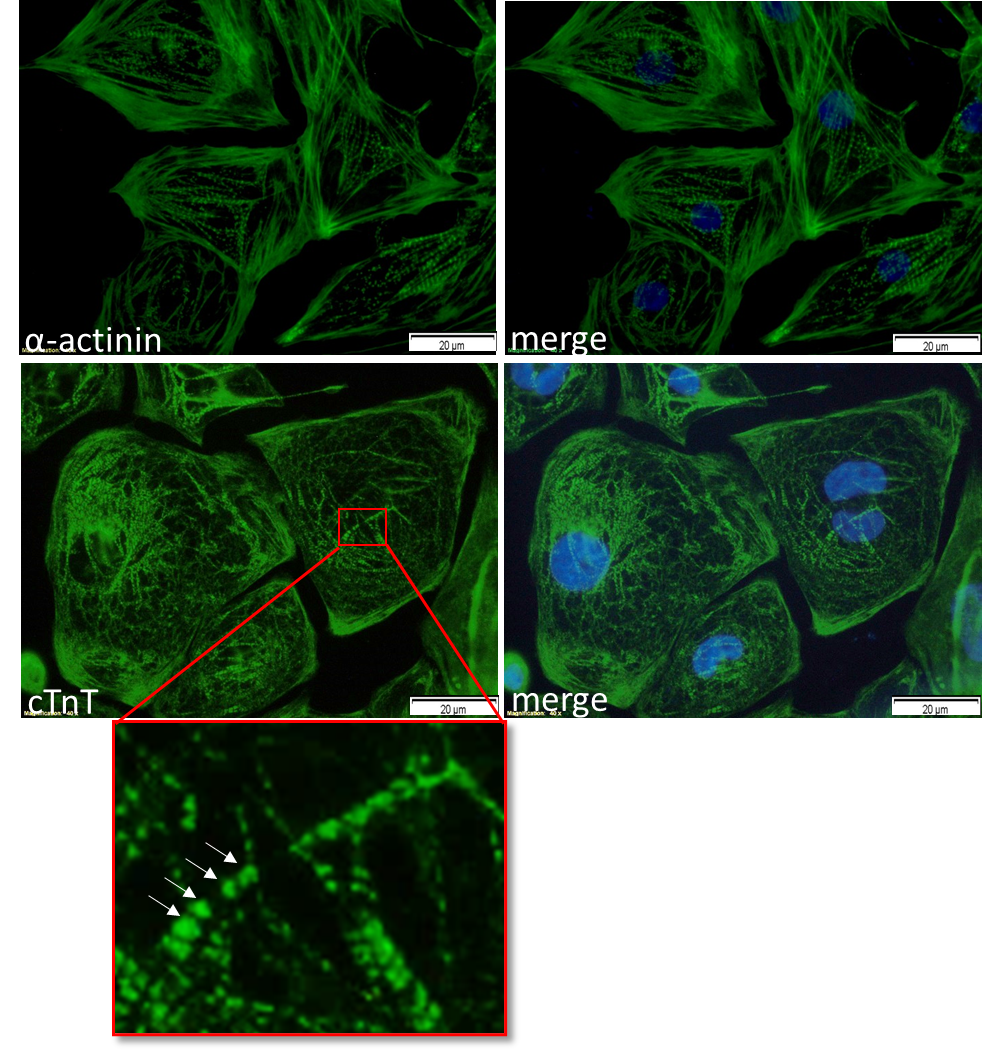


Figure S 3 Structural characterization of cardiomyocytes generated from iPSCs. Immunostaining at day 30 for α-actinin (green) and cTnT (green) shows sarcomere organization. Nuclei were stained with DAPI (blue). Scale bar: 20 μm.


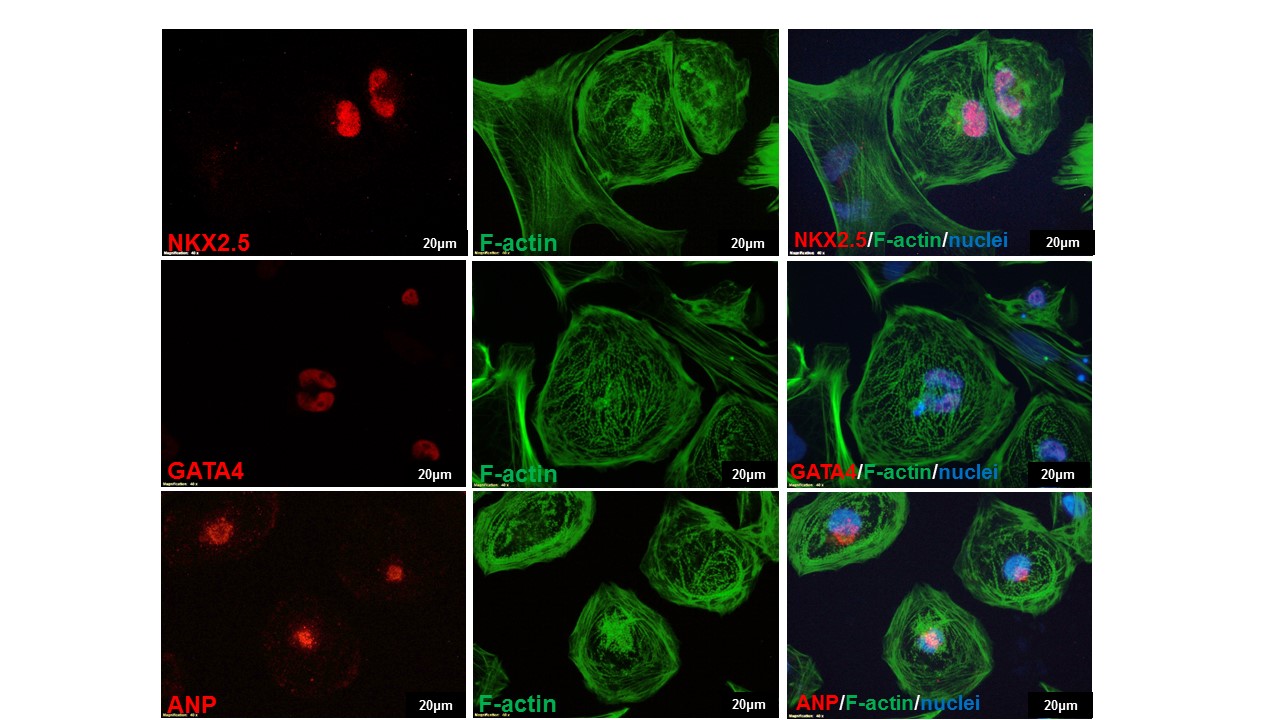


Figure S 4 Generations of iPSC-CMs were verified via immunofluorescence staining of cardiomyocytes markers: transcription factor NKX2.5 (red), transcription factor GATA4 (red) and natriuretic peptide A (ANP, red) at day 30. Nuclei were stained with DAPI (blue). Scale bar: 20 μm.


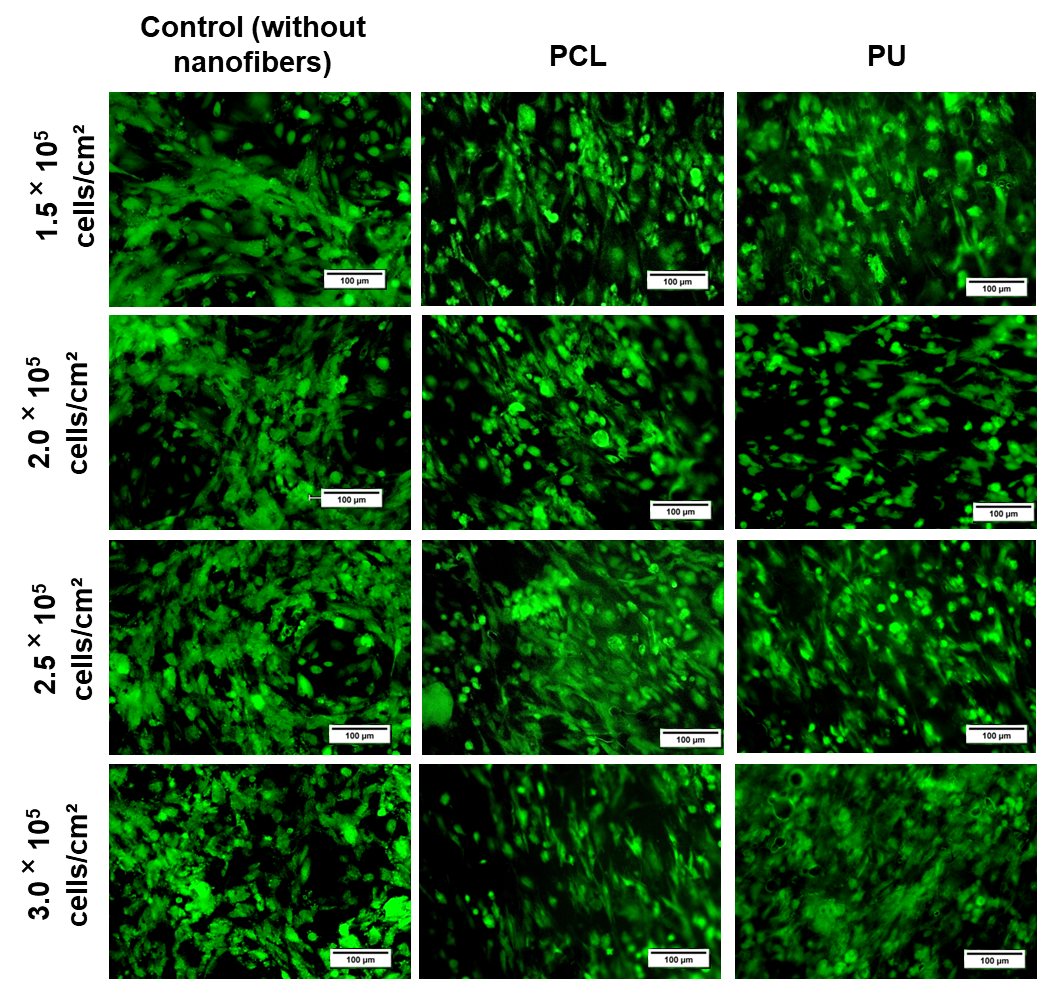


Figure S 5 The iPSC-CMs stained with calcein-AM (CAM) (green color) were cultured for 72 h on different nanofibrous mats depending on cell seeding density in 20x magnification.
