# Supplementary material for: Maturation of human cardiomyocytes derived from induced pluripotent stem cells (iPSC-CMs) on polycaprolactone and polyurethane nanofibrous mats
Source: Sci Rep. 2024 Jun 5;14:12975. doi: 10.1038/s41598-024-63905-z (PMC11153585; doi:10.1038/s41598-024-63905-z)
Supplement: Supplementary file 2 — Supplementary Movie 1. [file 41598_2024_63905_MOESM2_ESM.docx]

Movie 1. Spontaneous contraction of iPSC-CMs after differentiation.
